# Learning while doing: program evaluation of the Medical Library Association Systematic Review Project

**DOI:** 10.5195/jmla.2018.286

**Published:** 2018-07-01

**Authors:** Catherine Boden, Marie T. Ascher, Jonathan D. Eldredge

**Affiliations:** Associate Librarian, Leslie and Irene Dube Health Sciences Library, University of Saskatchewan, Saskatoon, SK, Canada; Lillian Hetrick Huber Endowed Director, Health Sciences Library; Assistant Professor, School of Medicine; and Assistant Professor in Epidemiology and Community Health, School of Health Sciences and Practice; New York Medical College, Valhalla, NY; Associate Professor and Evidence Based and Translational Sciences Collaboration Coordinator, Biomedical Informatics Research, Training and Scholarship Unit, Health Sciences Library and Informatics Center, University of New Mexico, Albuquerque, NM

## Abstract

**Objectives:**

The Medical Library Association (MLA) Systematic Review Project aims to conduct systematic reviews to identify the state of knowledge and research gaps for fifteen top-ranked questions in the profession. In 2013, fifteen volunteer-driven teams were recruited to conduct the systematic reviews. The authors investigated the experiences of participants in this large-scale, volunteer-driven approach to answering priority research questions and fostering professional growth among health sciences librarians.

**Methods:**

A program evaluation was conducted by inviting MLA Systematic Review Project team members to complete an eleven-item online survey. Multiple-choice and short-answer questions elicited experiences about outputs, successes and challenges, lessons learned, and future directions. Participants were recruited by email, and responses were collected over a two-week period beginning at the end of January 2016.

**Results:**

Eighty (8 team leaders, 72 team members) of 198 potential respondents completed the survey. Eighty-four percent of respondents indicated that the MLA Systematic Review Project should be repeated in the future and were interested in participating in another systematic review. Team outputs included journal articles, conference presentations or posters, and sharing via social media. Thematic analysis of the short-answer questions yielded five broad themes: learning and experience, interpersonal (networking), teamwork, outcomes, and barriers.

**Discussion:**

A large-scale, volunteer-driven approach to performing systematic reviews shows promise as a model for answering key questions in the profession and demonstrates the value of experiential learning for acquiring synthesis review skills and knowledge. Our project evaluation provides recommendations to optimize this approach.

## INTRODUCTION

Health sciences librarians have played integral roles in developing and supporting systematic reviews over the past 30 years. Currently, health sciences librarians often are considered to be essential members of teams that implement systematic reviews [1]. Health sciences librarians have traditionally contributed literature search expertise to health-related systematic reviews, although they increasingly contribute to other aspects of systematic reviews [2, 3]. Four percent to 80% (depending on the role) of North American health sciences librarians serve roles such as project manager or leader, research question developer, critical appraiser, data extractor, report writer, and disseminator [4].

Conducting systematic reviews means, for many health sciences librarians, adding a new set of skills to their repertoire. For busy librarians, the possibility of developing new skills in a supportive environment by working on research that addresses professional priorities [5] is appealingly efficient. This paper describes an evaluation of the Medical Library Association (MLA) Systematic Review Project (hereafter, MLA SR Project) to better understand the benefits and challenges of a large-scale distributed model for addressing a profession’s research priorities, while growing its research capability.

The MLA SR Project, comprising fifteen separate teams, represents the latest phase of a decade-long effort. MLA released its renewed research agenda, *The Research Imperative,* in 2007 [6, 7]. Inspired by opportunities for applying research in evidence-based practice, the new policy recommended that the MLA Research Section create a forum for identifying research priorities for health sciences librarianship. The MLA Research Section authorized members of the Research Agenda Committee (RAC) to conduct a delphi study in 2008 of MLA leaders and researchers to identify the most important and answerable research questions [8, 9]. In 2011, the Research Section authorized a second delphi study with a far more ambitious reach in terms of recruiting more leaders and researchers and correcting question redundancy in the first study [10].

The RAC made two observations based on their analysis of the 2011 fifteen top-ranked questions that were produced by the delphi technique [11]. First, they noted that a number of questions reflected anxieties about the future of health sciences libraries during a recession that the 2008 delphi study had barely detected. Second, most questions could fit within six major categories adapted from two content analyses of research in librarianship: collections, education of users, information access, outcomes or impact, professional issues, and value [12, 13]. Some questions were wide-ranging: “What is the quantifiable evidence that the presence of a librarian, not just information resources, improves patient outcomes, increases research dollars, improves student outcomes (e.g., better board scores), or increases hospital intelligence?” Other questions were more focused: “What are the most effective instructional methods for teaching informatics, knowledge management, or evidence-based practice in health sciences curricula?”

RAC members proposed to the MLA Research Section Executive Committee that the top questions be answered so that practitioners would have the best available research evidence when making important decisions. Systematic reviews provide this needed highest level of evidence. The RAC and other MLA leaders also were disappointed that researchers did not pursue any of the high-priority research questions generated during the previous 2008 delphi process.

Systematic reviews additionally can assess the existing research evidence and point to areas where researchers could focus their attention productively. Thus, RAC members decided to link the new fifteen top-ranked questions proactively to envisioned systematic reviews to produce answers and identify the state of knowledge and research gaps in each area [14]. RAC members developed a protocol to guide forming and monitoring the fifteen nearly completely autonomous teams [15]. This process might be the first time a professional association has linked its research agenda to a systematic review process [16].

Almost 200 librarians and informationists from across the globe responded to a call for volunteers. Teams were assigned based on reported question preferences and began work on the systematic reviews in March 2013. While each team worked autonomously, team leaders met periodically with RAC members to share progress and experiences. Teams’ membership compositions have fluctuated since project inception due to volunteers’ other professional and personal demands. Leadership changes for 3 teams have entirely impeded these teams’ progress. An MLA SR Project status report from January 2017 indicated that 3 teams had completed their reviews and published articles (one of which was awarded the 2015 MLA Ida and George Eliot Prize [17]), 8 teams were continuing to make significant progress, and 4 teams were in very early stages or needed to restart [18]. At least 1 team elected to conduct a scoping review instead of a systematic review due to the emerging status of the research question.

The MLA SR Project exhibits uniqueness in its scale of almost 200 volunteer librarians and its scope of 15 systematic reviews on high-priority questions generated by MLA leaders. The current study is a program evaluation designed to: (1) investigate the experiences of librarians participating in this large-scale, volunteer-driven approach to answering priority research questions by surveying all volunteers and (2) evaluate how well this approach fostered professional growth for health sciences librarians wishing to acquire systematic review knowledge and skills.

## METHODS

### Recruitment

All MLA SR Project volunteers were invited to participate in a survey via email using the most current email address available. Responses were collected over a two-week period beginning at the end of January 2016. Reminder emails were sent one week and one day prior to the survey closing date.

Immediately prior to survey distribution, all team leaders were asked to provide current email addresses and the status (active, withdrew, never active) for each of their team members. Nine of fifteen team leaders responded; thus, up-to-date information about the team members was not available for six teams. For teams whose updated information was not available, the authors used contact information from the original volunteer roster.

Team leaders were also asked to inform their team members about the survey. The initial team rosters in 2013 listed 15 teams with a total of 199 librarians volunteering for the project. At the time of the survey, the authors estimated that 14 librarians withdrew before their projects began and 20 librarians withdrew at an early stage of their projects. Volunteers were invited to participate in the survey regardless of their status, so the survey was distributed to 198 individuals (1 team leader was an author of this article and, thus, did not complete the survey). The University of Saskatchewan Behavioral Research Ethics Board determined this program evaluation survey to be exempt from review.

### Survey instrument

The eleven-item survey elicited respondents’ experiences about scholarly and non-scholarly outputs, project successes and challenges, lessons learned, and future directions (supplemental appendix). The investigators requested respondent characteristics (e.g., role on the project).

### Analysis

Frequency statistics (counts, percentages, averages) were calculated for responses to multiple-choice questions. Short-answer questions (except the communication outputs question) were analyzed using thematic analysis with an inductive approach to identifying themes, following the recommendations of Braun and Clark [19]. Two reviewers independently coded the data and identified themes. Three authors reviewed, discussed, and achieved agreement on the identified themes.

We collated responses to the question about outputs (“Please list all publications, presentations, posters, blogs, tweets, Facebook posts, and Snapchat stories or other ways that you have communicated either your team’s experiences or systematic review results to the profession”). We removed duplicate outputs and identified types of communication outputs: journal articles, conference presentations or posters, other presentations, and social media. The exact number of unique outputs could not be calculated because it was not possible to ensure that an output was unique, as respondents were anonymous and multiple members of a team might report duplicate outputs; that is, all team members might independently report an article that they published together. These data were captured in the 2017 status report published in *Hypothesis* [18].

## RESULTS

Eighty (8 team leaders, 72 team members) of 198 potential respondents completed the survey (40% response rate). Twenty-two percent of the 80 respondents were on teams that had completed their reviews; 53% (30% contributing fully, 23% contributing intermittently) were on teams that still had reviews in progress; 5% were on teams that were restarting with a new team leader; 16% were not able to work with the team to completion; and 4% left this question blank.

Reasons for withdrawal that were selected from the provided options (respondents indicated all that applied) included: lack of capacity due to other work commitments (n=8); the project took longer than expected (n=6); change of job (n=2); and personal reasons (n=1). Other reasons for early withdrawal (“Other, please specify” option) included: personal reasons (e.g., “parental leave”); frustration (e.g., “Frustration with our group’s logistical application of the SR methodology”); poor communication (e.g., “No one contacted us after a few months”); and lack of guidance and organization (e.g., “Poorly organized and lack of centralized tools”).

For the overall population of 198 participating librarians, we roughly estimate that 15% were on teams that completed a review, 20% were on teams that had a new team leader, and 65% were on teams that were still working on their reviews. Our sample was representative of the overall proportions of team leaders and team members who either dropped out early or were able to continue.

Team members have communicated their experiences and systematic review results to the profession via standard scholarly routes, social media, and networking with colleagues. Most respondents indicated that their teams produced one or more of the following: journal articles (published or submitted), conference presentations and posters, and other presentations (e.g., librarian forums, workshops). Presentations and posters were shared globally (United States, Canada, United Kingdom, France, South Africa, and Ireland), reflecting the international makeup of the project membership. Teams have also communicated their experiences and the results of their work via social media (Twitter, Research Gate, blogs, and Google Plus*). Two teams have received awards for their work.

A majority of respondents (84%) indicated that the MLA RAC should consider repeating this initiative in the future, either to both answer research questions that are relevant to the profession and facilitate librarian learning of systematic review methods (66%), answer questions relevant to the profession only (10%), or facilitate librarian learning about systematic review methods only (8%). Ten percent indicated that the MLA RAC should not consider repeating this initiative in the future, and 6% did not provide a response. Most respondents (84%) were interested in participating in another systematic review (8% as a literature search expert only, 76% in all aspects of the systematic review).

**Figure 1:**
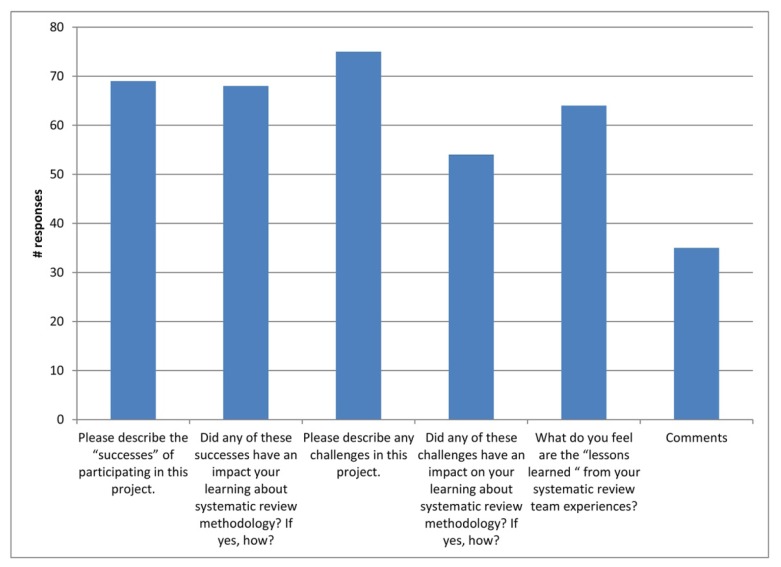
The number of respondents providing answers to the survey questions evaluated in the thematic analysis

Thematic analysis of participant responses to the six short-answer questions (i.e., questions 3–6, 8, and 10) yielded five broad themes: learning and experience, interpersonal (networking), teamwork, outcomes, and barriers. The number of respondents contributing to each question included in the thematic analysis is provided in Figure 1.

### Thematic analysis

#### Theme 1: learning and experience

##### Hands-on

Librarians described hands-on, practical learning about systematic (or scoping) review methods. For some, this represented a mechanism for professional growth and, for others, a new research method. For instance, a respondent indicated:

It was the “learning by doing” aspect that was the greatest value added of this experience. Intimately understanding proper reporting guidelines, protocols, bias, data abstraction—in short all the steps and nuisances of completing a systematic review (or other types of literature reviews).

##### Practical skills

Respondents learned new tools and practical skills, such as:

I had taken a couple of different [continuing education courses] CEs in the systematic review methodology, but going through the process myself cemented the process and I really had some lessons learned coming out of this project. For example, piloting inclusion and exclusion criteria as a team before delving deeply into the review.

##### Complexity of systematic reviews

Participants reflected on the complexity and time intensiveness of the systematic review process and gained insight into the “investigator” perspective. The act of participating in a review team revealed the complexities of team-based research. As one participant noted:

Learning about the process of a systematic review from the perspective and role of the investigator was invaluable. I have a better understanding of the process of a systematic review and how time-intensive it is to complete one. Going through the physical process of choosing relevant abstracts was time-consuming, but incredibly thought-provoking. Then meeting as a group to decide a select few that did not meet consensus was interesting as well.

Another said:

It illustrated how complicated it is to work in a large group, the importance of communication throughout the process.

##### Leadership skills

Team leaders described learning to lead a group of geographically dispersed librarians through a complex research project. As one leader noted, “This was the first time I had worked in a large team to produce a systematic review so there was a development in my team work and leadership skills over the course of the project.” Where the team dynamics or leadership were strong, teamwork fostered learning, knowledge-sharing, and the “power” of a team of librarians. For example, “Being somewhat new to MLA and the medical librarian profession, it was a fantastic experience to learn how powerful a group of medical librarians can be.” However, teams were not uniformly effective, and some individuals learned “what not to do” through more challenging team experiences, such as “Learning about difficulties to deal with every team member and acquiring skills to get around those difficulties.”

#### Theme 2: interpersonal (networking)

The international make-up of teams promoted networking beyond the United States and presented opportunities for introducing international perspectives. As one participant wrote, “It’s been informative to discuss and understand an international perspective to the topic. We have increased our network of colleagues and contacts.” Participation on the project also gave newer systematic reviewers contacts for future support. As a participant stated, “I gained a point of contact (e.g., our team leader) for any systematic review related question.”

#### Theme 3: teamwork

##### Team dynamics

Team composition and dynamics significantly impacted progress and the experience of the project. As an example, one team member wrote:

The challenges faced were not unlike any other group project one might be a part of. People came with different working styles, personalities, and varying degrees of time and resources to dedicate to the project.

Although team diversity, workload equity, and the impact of external factors (e.g., personal commitments, family issues, professional issues) challenged team interactions, teamwork also allowed knowledge-sharing and learning from colleagues. For instance, one respondent wrote “TEAMWORK!!! We all learned so much from each other!”

##### Communication

Participants expressed communication challenges in terms of quantity, quality, and technical capacity. Large, geographically dispersed teams with team members in a variety of settings, some of which did not allow certain types of communication technologies, contributed to communication challenges; for instance, “hospital firewalls blocked most free communication tools.” While the global reach of the project was appreciated for networking, it was perceived as hampering communication. Respondents mentioned challenges with time zones, such as, “We also started out with 12+ members across 4 time zones (including UK) making it difficult to find good times to meet and make decisions by consensus.”

##### Leadership and value of a good leader

For some teams, communication from the team leaders was perceived as inadequate, or the absence of the team leader left the team members confused about the status of the project:

Very intermittent communication, and what there was often primarily directive: “I did this, now you do that.” Our project still isn’t finished and until last week, I hadn’t heard anything about it in close to a year; I just assumed it had died on the vine.

Recognizing the key and demanding role of the team leader, some participants suggested co-leaders:

The team leader should be someone that knows they’ll be able to commit the kind of time that that is required for managing an international team on a project like this. Perhaps co-leads would be a good idea?

##### Project management

Team members looked to team leaders for training, project management, and methodological expertise. As one respondent wrote, “Our Team Leader set us up for success; she had all the necessary tools and information to guide the team through the process.” Conversely, another participant reported that one challenge to learning was a lack of team leader knowledge: “Frustration with team leader’s level of knowledge of the methodology, some surprise at varying levels of search skills.” Team leaders had a pivotal and complex role that was critical to team success and progress when team leaders were effective and detrimental when team leaders lacked time, experience, or project management skills. Faced with large, distributed teams, project leaders had to manage time, a volunteer workforce, and technology. Achieving timelines through accountability of team members to deadlines was challenging for team leaders:

Members also have wide variety in the amounts of time they can/will put in on the project. This latter point places me in a precarious position—I need all of them to stay on the project and do the work, but feel like I need to avoid setting tough deadlines for fear some will drop out. So it’s taken a looooong time to get things done. That in turn has frustrated others, who get their work done relatively quickly but wonder if this project is ever going to be done.

Team members wanted clear deliverables, deadlines, and accessible workflows (e.g., through use of tools and technology); for instance, “Having good workflows, technologies and systems in place which are easy to use and easy to access for all team members. Overall communication and regular meetings are absolutely essential.”

#### Theme 4: outcomes

##### Publications and presentations

Publishing the systematic review results and winning an MLA award was a significant success for members of one team: “I think it was great to get that positive return of publication and know that at least for the one I was part of that our methodology was sound enough and our work thorough enough that it merited getting published.” Even teams whose systematic review was still underway had posters and presentations at conferences.

##### Implications for professional practice

Respondents felt that their participation had implications for professional practice, as it boosted their credibility, confidence, and knowledge in providing systematic review support and training. For some, it added a method to their research toolbox. For others, the experience provided insight into the “investigator” experience, which deepened their understanding of faculty and student systematic review support needs:

Intimately understanding proper reporting guidelines, protocols, bias, data abstraction—in short all the steps and nuisances of completing a systematic review (or other types of literature reviews). A huge impact was being able to demonstrate that knowledge among researchers, faculty members and other colleagues and be able to back that up at every step.

Collaboration and research products (i.e., publications and presentations) contributed to evidence for tenure and promotion and exposure of librarians in a wider forum. A new librarian valued the “Increased exposure at the national level (I’m a new librarian with 4.5 years experience and only 2 years when I started this project)…[I]tems on my CV for promotion and tenure.”

#### Theme 5: barriers

##### Lack of training and support infrastructures or tools

Respondents expressed a desire for MLA to provide funding, training, or communication venues (e.g., email discussion lists, protected time at conferences) to support the systematic review teams. For example, one respondent wrote:

If MLA do[es] this again it would be useful to have a small budget associated with each project to facilitate working and publication (there are costs associated with many “open access” publication routes).

##### Knowledge levels of participants

A lack of experience with systematic review methods (generally or in specific aspects of the review process) and, sometimes, a lack of background with other research that the systematic review topic required challenged teams. As one respondent put it:

Our team had very few people with any experience doing systematic reviews and I don’t feel like a project like this (large team, geographically dispersed, not straight-forward question) was the best way to teach those less experienced.

##### Logistics of and barriers caused by geographic dispersion

Geographic dispersion created difficulty in terms of groups connecting and willingness to participate in a project with people they did not know and who were not in close geographic proximity. For example, “Working with people all over the world...very difficult to coordinate some things, hard to maintain a sense of connection.” Some respondents reported a preference for working with “a group of people living in the same area because this would make possible and easier for meeting in person and sharing information more easily.”

##### Research question

Some questions from the research agenda did not lend themselves to a systematic review. Teams struggled with the ambiguity of the research questions and with refining the research questions to better suit either systematic or scoping review methodology.

I think the ambiguity of our research question has led to several challenges. The various concepts in our question can have different interpretations, which made coming to a common understanding challenging. As a result, our interpretation of the question and search were very broad, our result set huge, our screening time long, etc.

## DISCUSSION

The MLA SR Project is a large, distributed project aiming to address research priorities for health sciences librarians, while also training librarians to conduct systematic reviews or, in one team’s case, a scoping review. One goal in conducting this program evaluation is to investigate the experiences of volunteers in this large-scale, volunteer-driven approach to answering key questions in the profession. A part of this question is whether the teams are achieving success in completing their reviews. The short answer to this question is a qualified “yes,” but slowly. There are successes that reveal potential for this kind of initiative, including peer-reviewed articles [17, 20, 21], awards, conference paper or poster presentations, and sharing via social media. Most teams have completed or are well on their way to completing their reviews. While this project evaluation was conducted three years after teams initiated their work, systematic reviews, particularly those consisting of far-flung networks of volunteers, take considerable time. Our survey results reflect that reality.

We were also interested in how well the initiative fostered professional growth for health sciences librarians who want to develop systematic review knowledge and skills. There is clear evidence that librarians learned new skills as a direct result of participating on a systematic review team, although the quality of learning experiences varied. As for practical knowledge and skills gained, librarians participating in the initiative reported an increased understanding of the complexity of systematic reviews from librarian and researcher perspectives, understanding of teamwork and collaboration in research, and development of leadership or project management skills. Other benefits of participation included networking (thereby developing a support system for future learning and collaboration), opportunities to gain international perspectives on the topics, and scholarly publications, paper or poster presentations, and awards. As testament to success, a majority of participants (84%) indicated that the MLA RAC should consider repeating this initiative in the future and indicated an interest in participating again.

In the context of a workforce needing to acquire new skills to meet the demand for synthesis review services [2, 3], such large-scale training is a significant contribution to the profession. Some participants also valued involvement in a systematic review team to develop or deepen research skills (i.e., research capability development). To be clear, providing training was not a formal requirement of the teams. Each team approached systematic review knowledge and skill development in its own way. The approach to training for the overall project, therefore, cannot be accurately described in a single formal approach, such as action learning (e.g., Booth et al. [5], Mumford [22]) or mentoring (e.g., Eldredge [23], Fyfe and Dennett [24], Lorenzetti and Powelson [25], Ritchie and Genoni [26]).

The training approaches of the MLA SR Project teams might be loosely described as learning-by-doing or experiential learning, which likely incorporates elements from a variety of learning mechanisms whose composition varied across teams. Studies of professional development combining experiential learning with work-related tasks [5, 27] and our own project evaluation provide preliminary evidence that a “learning by doing” approach is worth evaluating more thoroughly as a mechanism for professional development.

As with any new initiative, not everything went as expected for team members. Workload management issues, a desire for additional training and infrastructure support, and disparities in team members’ experiences reportedly affected progress. Geographic dispersion, the research questions, and team leader experience were instrumental (positively and negatively) to team dynamics and outcomes. The research questions were provided to team leads directly from the delphi study [28]. Team leaders were tasked with restating the question so that they were answerable by a systematic review. Some teams either disregarded this direction or chose different approaches, possibly because this expectation was not clear. As a consequence, some teams struggled with their research questions. Respondents’ comments highlighted the importance of a team leader with knowledge of systematic reviews methods, good leadership or project management skills, and effective communication.

Future implementations of this initiative could be optimized. Clear guidance on the requisite experience could help potential team leaders and members select roles. The RAC might also request information about specific skills and knowledge from volunteers to facilitate the allocation of volunteers to teams, particularly team leader positions. Team success depends on a variety of skills and knowledge beyond systematic reviews methods, such as technical skills (e.g., systematic review tools), research team dynamics, project management, communication, experience on a research team, and time management. The RAC explicitly indicated that the review teams were to proceed autonomously. Yet, the RAC did provide recurring opportunities for team leaders to meet with them to discuss their projects, while also filling team leaders’ requests for guidance. Augmentation of this knowledge-sharing among team leaders would generate a sense of shared purpose and support.

This project evaluation has limitations. It was not possible to directly compare the characteristics of respondents to nonrespondents. However, our estimates suggest that the sample was reasonably representative, although individuals from teams that lost their team leaders and had to restart might be slightly underrepresented in our sample. While we believe the results reflected the experiences of the MLA SR Project participants, this was an evaluation of a specific initiative. The survey was not designed to be generalizable to other instances of learning by doing or implementations of professional associations’ research agendas.

Although the particularities of this initiative might not be generalizable, we believe the results of the survey will interest many health sciences librarians and informatics practitioners. Some of the recommendations might inform the development of similar initiatives. For the future, we recommend: (1) manage expectations, boundaries, and levels of commitment on an ongoing basis; (2) establish strong communication routes through and beyond project leaders to facilitate communication to all members; and (3) assign team membership with an eye toward achieving a balance of expertise (subject knowledge and methodological expertise) and strong leadership. Our results show that the MLA SR Project was valued as a learning opportunity. Prospectively designed studies could provide more detailed evaluation of the impact of systematic review experience on team dynamics and success. In the future, a coordinated educational experience could be designed and implemented to amplify experiential learning and provide a more uniformly positive opportunity for professional development.

## SUPPLEMENTAL FILE

Appendix Survey questions

## Footnotes

* The Google+ community is available at <https://plus.google.com/u/0/communities/115832551443909297773?cfem=1>.
