# Supplementary material for: Learning while doing: program evaluation of the Medical Library Association Systematic Review Project
Source: J Med Libr Assoc. 2018 Jul 1;106(3):284–93. doi: 10.5195/jmla.2018.286 (PMC6013139; doi:10.5195/jmla.2018.286)
Supplement: Appendix [file jmla-106-284-s001.pdf]

JMLA Journal of the Medical Library Association

SC

Supplemental content to
J Med Libr Assoc. Jul;106(3):dx.doi.org/10.5195/jmla.2018.286
www.jmla.mlanet.org
© Boden, Ascher, Eldredge 2018

Learning while doing: program evaluation of the Medical Library Association Systematic Review Project

Catherine Boden; Marie T. Ascher; Jonathan D. Eldredge, AHIP

APPENDIX

Survey questions

1. What is your current status on this project?
   - Our team has completed our review.
   - Our team is still working on the review, and I am still able to contribute to the team fully.
   - Our team is still working on the review, and I am still able to contribute to the team intermittently.
   - Our team has not completed the review and is just restarting again under a new leader.
   - I was not able to work with my systematic review team until completion.
     Reason (choose all that apply):
     - Lack of capacity due to other work commitments
     - Lack of interest
     - Change of job
     - The project took longer than expected
     - Personal reasons
     - Other, please specify
   - Our team has just formed, and we are just starting the review.

2. Please list all publications, presentations, posters, blogs, tweets, Facebook posts, and Snapchat stories or other ways that you have communicated either your team's experiences or systematic review results to the profession.

3. Please describe the "successes" of participating in this project.

4. Did any of these successes have an impact on your learning about systematic review methodology? If yes, how?

5. Please describe any challenges in this project.

6. Did any of these challenges have an impact on your learning about systematic review methodology? If yes, how?

7. Having participated in this project, are you interested in conducting another systematic review, either on a library and information science (LIS) topic or on a health sciences topic in the future?
   - Yes, but as a literature search expert only.
   - Yes, in all aspects of the systematic review.
   - No.

8. What do you feel are the "lessons learned" from your systematic review team experiences?

SC
Supplemental content to
J Med Libr Assoc. Jul;106(3):dx.doi.org/10.5195/jmla.2018.286
www.jmla.mlanet.org
© Boden, Ascher, Eldredge 2018

9. Do you feel that the MLA Research Agenda Committee should consider repeating this program in the future?
   - No.
   - Yes, for answering research questions relevant to our profession.
   - Yes, for facilitating health sciences librarians to learn systematic review (SR) methodology.
   - Yes, for answering research questions relevant to our profession and to learn SR methodology.

10. Comments

11. Are you (or were you) a team leader?
   - Yes.
   - Yes, but I was not able to work with my team until completion.
   - Not initially, but I later became a team leader.
   - No.
